# CXCL12-induced neurotoxicity critically depends on NMDA receptor-gated and l-type Ca^2+^ channels upstream of p38 MAPK

**DOI:** 10.1186/s12974-016-0724-2

**Published:** 2016-09-23

**Authors:** Ana B. Sanchez, Kathryn E. Medders, Ricky Maung, Paloma Sánchez-Pavón, Daniel Ojeda-Juárez, Marcus Kaul

**Affiliations:** 1 Infectious and Inflammatory Disease Center, Sanford Burnham Prebys Medical Discovery Institute, 10901 North Torrey Pines Road, Bldg. 10, La Jolla, CA 92037 USA; 2 Department of Psychiatry, University of California, San Diego, 9500 Gilman Drive, San Diego, CA 92093 USA; 3 Present address: UC San Diego Health, 200 W. Arbor Drive #8765, San Diego, CA 92103 USA

**Keywords:** CXCL12, CXCR4, Neurotoxicity, Cell death, Calcium channel, Inhibitors, p38 MAPK, Kinase activity, Immunofluorescence microscopy

## Abstract

**Background:**

The chemokine receptor CXCR4 (CD184) and its natural ligand CXCL12 contribute to many physiological processes, including decisions about cell death and survival in the central nervous system. In addition, CXCR4 is a co-receptor for human immunodeficiency virus (HIV)-1 and mediates the neurotoxicity of the viral envelope protein gp120. However, we previously observed that CXCL12 also causes toxicity in cerebrocortical neurons but the cellular mechanism remained incompletely defined.

**Methods:**

Primary neuronal-glial cerebrocortical cell cultures from rat were exposed to a neurotoxicity-inducing CXCL12 concentration for different times and the activity of the stress-associated mitogen-activated protein kinase p38 (p38 MAPK) was assessed using an in vitro kinase assay. Neurotoxicity of CXCL12 and cellular localization of p38 MAPK was analyzed by immunofluorescence microscopy. Pharmacological inhibition of NMDA-type glutamate receptor-gated ion channels (NMDAR) of l-type Ca^2+^ channels was employed during 12- and 24-h exposure to neurotoxic amounts of CXCL12 to study the effects on active p38 MAPK and neuronal survival by Western blotting and microscopy, respectively. Neurotoxicity of CXCL12 was also assessed during pharmacological inhibition of p38 MAPK.

**Results:**

Here, we show that a neurotoxic amount of CXCL12 triggers a significant increase of endogenous p38 MAPK activity in cerebrocortical cells. Immunofluorescence and Western blotting experiments with mixed neuronal-glial and neuron-depleted glial cerebrocortical cells revealed that the majority of active/phosphorylated p38 MAPK was located in neurons. Blockade of NMDAR-gated ion channels or l-type Ca^2+^ channels both abrogated an increase of active p38 MAPK and toxicity of CXCL12 in cerebrocortical neurons. Inhibition of l-type Ca^2+^ channels with nimodipine kept the active kinase at levels not significantly different from baseline while blocking NMDAR with MK-801 strongly reduced phosphorylated p38 MAPK below baseline. Finally, we confirmed that directly blocking p38 MAPK also abrogated neurotoxicity of CXCL12.

**Conclusions:**

Our findings link CXCL12-induced neuronal death to the regulation of NMDAR-gated ion channels and l-type Ca^2+^ channels upstream of p38 MAPK activation.

## Background

The chemokine receptor CXCR4 (CD184) and its physiological ligand stromal cell-derived factor (SDF)-1/CXCL12 are both widely and constitutively expressed throughout the mammalian organism and play important biological roles in development and tissue homeostasis [1–4]. As such, the CXCR4/CXCL12 receptor-ligand system is involved in many physiological processes beyond chemotaxis, including embryogenesis, vascularization, brain development, neurogenesis, hematopoiesis, inflammation, tissue repair, and cancer [2, 4–7]. While cell migration along the chemokine gradient contributes in most of these processes, the functions of CXCR4 and CXCL12 in the central nervous system (CNS) also include the regulation of axonal outgrowth and patterning, synaptic function, and plasticity as well as decisions about cell death and survival (reviewed in [4–6, 8, 9]). One of the best recognized pathological function of CXCR4 is its role as a co-receptor during infection with human immunodeficiency virus (HIV)-1 and the ability of CXCL12 to interfere in the infection process, at least in vitro [10, 11]. Furthermore, we and others have shown that CXCR4 can mediate the induction of neuronal injury and apoptosis by HIV-1 envelope protein gp120 [12–16], a neurotoxic process that downstream of chemokine receptor engagement also involves excessive excitatory activation of the *N*-methyl-*d*-aspartate type of glutamate receptors (NMDAR) [17]. HIV-1 infection can result in proteolytic cleavage of CXCL12, and the truncated chemokine exerts neurotoxicity through a CXCR3-mediated pathway [18]. However, investigating whether CXCL12 can prevent a CXCR4-using HIV-1 gp120 from inducing neuronal apoptosis, we observed that full-length CXCL12 itself is toxic to terminally differentiated cerebrocortical neurons via a pathway that depended on CXCR4 and implicated p38 mitogen-activated protein kinase (p38 MAPK) [12, 13]. We also found that exposure to CXCL12 triggered a transient increase of intracellular Ca^2+^ in cerebrocortical neurons [13]. Others have linked neurotoxic CXCR4 activation to astrocytic release of glutamate which in turn was stimulated via a pathway involving tumor necrosis factor (TNF)α, prostaglandins, and microglial activation [19]. The release of astrocytic glutamate suggested that neuronal glutamate receptors and excitotoxicity may contribute to CXCR4-mediated neurotoxicity. Neuronal excitotoxicity is associated with excessive influx of extracellular Ca^2+^ resulting in intracellular Ca^2+^ overload, downstream activation of p38 MAPK and p53, release of cytochrome c and other molecules from mitochondria, such as apoptosis-inducing factor, activation of caspases, free radical formation, lipid peroxidation, chromatin condensation, and eventually apoptosis [20, 21]. Considering the importance of Ca^2+^ in excitotoxicity, it was therefore not surprising that inhibitors of Ca^2+^ ion channels, including NMDA-type glutamate receptor-gated ion channels (NMDAR), were found to prevent toxicity of HIV gp120 [19, 22, 23]. Here, we show that blockade of l-type Ca^2+^ channels and NMDAR both prevent CXCR4-mediated toxicity of CXCL12 in cerebrocortical neurons. We also demonstrate that a neurotoxic concentration of CXCL12 increases the amount of active, phosphorylated p38 MAPK, which is primarily located in neurons. Moreover, we find that inhibition of l-type Ca^2+^ channels and NMDAR limits or diminishes the activation of the stress kinase in neurons. Finally, we confirm that directly blocking p38 MAPK also abrogates neurotoxicity of CXCL12. Hence, both types of Ca^2+^ channels appeared to control CXCL12-induced neuronal death upstream of p38 MAPK.

## Methods

### Primary rat cerebrocortical cell cultures

Mixed neuronal-glial cerebrocortical cell cultures, containing neurons, astrocytes, and microglia, were prepared from E16 Sprague-Dawley rat embryos (Harlan Sprague Dawley Inc., San Diego, CA) as previously described [12, 13, 24, 25]. Briefly, cerebrocortical cells from embryos were plated on poly-l-lysine-coated glass coverslips in 35-mm plastic dishes or 96-well plates for imaging (Falcon, BD Biosciences) and generally used for experiments after 17 days in vitro when the cultures contained ~30 % neurons, ~70 % astrocytes, and ~0.1 to 1 % microglia, and neurons were fully differentiated and sensitive to NMDA-induced excitotoxicity [12, 13]. To obtain glial cells, neurons were depleted by treating cerebrocortical cultures with 300-μM NMDA for 20 min 2 days prior to the experiment and preparation of cell lysate [24, 25]. All experimental procedures and protocols involving animals were in accordance with the NIH guidelines and approved by the Institutional Animal Care and Use Committee of the Sanford Burnham Prebys Medical Discovery Institute.

### Reagents

Recombinant CXCL12 (SDF-1β) was purchased from R&D Systems (Minneapolis, MN). Lyophilized CXCL12 preparations were reconstituted at micromolar (μM) concentrations in phosphate-buffered saline (PBS) containing 0.1 % BSA as carrier to 100 times the final concentration. Aliquots of reconstituted chemokine were stored at −80 °C until use [12, 13].

The non-competitive NMDAR antagonist MK-801 (dizocilpine maleate; (5R,10S)-(+)-5-methyl-10,11-dihydro-5H-dibenzo[a,d]-cyclohepten-5-10-imine, maleate) [26], the l-type Ca^2+^ channel blocker nimodipine (1,4-dihydro-2,6-dimethyl-4-(3′-nitrophenyl)-3,5-pyridinedicarboxylic acid 2-methoxyethyl-1-methylethyl ester) [27, 28], and the specific p38 MAPK inhibitor SB203580 [29] were purchased from EMD/Calbiochem (San Diego, CA) and reconstituted at 1000 times the final concentration in tissue culture-quality dimethyl sulfoxide (DMSO) (ATCC, Manassas, VA). Aliquots of reconstituted reagents were stored at −20 °C until use.

### Experimental treatment of cerebrocortical cell cultures

For experiments, cultures were transferred into pre-warmed Earle’s Balanced Salt Solution (EBSS) containing 1.8 mM Ca^2+^ and 5 μM glycine without additional Mg^2+^ or serum-free culture media. The cells were then incubated for 24 h in toxicity assays or for indicated time periods in kinase assay and Western blotting experiments in the presence or absence of MK-801 or nimodipine or SB203580 or CXCL12, or combinations thereof while the controls received vehicles only (BSA at 0.001 % and DMSO at 0.1 % final concentrations) as described before [12, 13, 25]. Following the incubation, the cultures were either fixed for immunofluorescence staining and assessment of neurotoxicity or lysed for protein kinase and Western blotting assays.

### Immunofluorescence staining and microscopy

The cerebrocortical cell cultures were fixed in 4 % paraformaldehyde (PFA) in PBS for 25 min at 4 °C and permeabilized with 0.2 TX-100. Neurons were immunolabeled with an antibody for neuron-specific microtubule-associated protein-2 (MAP-2; clone HM2, Sigma) and nuclei with the DNA dyes Hoechst 33342 or propidium iodide [12, 13, 24, 25]. For the investigation of cellular phospho p38 MAPK, the fixation of the cells in 4 % PFA was followed by ice-cold methanol for 10 min before permeabilization and immunostaining with anti-active p38 MAPK antibody (Ab) (Promega, WI) in combination with anti-MAP-2 [24] and labeling of nuclear DNA with the dye DRAQ5 (Thermo Fisher Scientific). Fluorescence microscopy was performed using a computer-controlled Axiovert 200-M microscope equipped with filters for DAPI, FITC, CY3, and CY5 (Zeiss) and a cooled CCD camera (Hamamatsu). Images were recorded and analyzed using the SlideBook software package (Intelligent Imaging Innovations, Denver, CO) as described earlier [13, 24, 30].

For the purpose of comparison, all samples of a given experiment were imaged under identical conditions, and the respective fluorescence channels were set to identical dynamic ranges in all images of the various experimental conditions. For better visualization, the fluorescence signal of DRAQ5 was shown in blue pseudocolor.

### Assessment of neurotoxicity

Neurotoxicity was assessed by fluorescence microscopy as previously described using a combination of immunostaining for neuron-specific MAP-2 and identification of pyknotic apoptotic nuclei after staining all nuclei with the DNA dyes Hoechst 33342 or propidium iodide [12, 13, 24, 25]. Neuronal survival was calculated using neuronal cell counts after subtracting MAP-2+ cells that displayed pyknotic or fragmented nuclei indicating apoptosis. The percentage of neurons was compared between the various experimental conditions and vehicle controls, which were defined as 100 % with regard to neuronal survival.

### Cell lysates, kinase assays, and immunoblotting

For the preparation of cell lysates for immunoblotting and protein kinase assays, cells were washed with ice-cold PBS and harvested in Triton X-100 Cell Lysis Buffer (20 mM Tris-HCl [pH 7.5], 150 mM NaCl, 1 mM Na_2_EDTA, 1 mM EGTA, 1 % Triton X-100, 2.5 mM sodium pyrophosphate, 1 mM beta-glycerophosphate, 1 mM Na_3_VO_4_, 1 μg/ml leupeptin; Cell Signaling Technologies; Beverly, MA) supplemented with 5 mM NaF (serine/threonine protein phosphatase inhibitor) and Complete Protease Inhibitor Cocktail (Roche; Indianapolis, IN) on ice for 10 min. The lysed samples were transferred into microcentrifuge tubes, sonicated four times for 5 s, and then cleared by centrifugation (13,200 x g, 10 min) at 4 °C. Lysate total protein concentrations were determined using the bicinchoninic acid (BCA) protein assay kit (Pierce/Thermo Fisher Scientific; Rockford, IL).

For immunoblotting, equal amounts of protein (usually 30 μg per lane) were separated by SDS-PAGE under reducing conditions and electroblotted onto polyvinyl difluoride (PVDF) membrane. After blocking remaining binding sites with BSA, the membranes were probed sequentially with anti-phospho p38 MAPK Ab (Promega, WI), total p38 MAPK Ab (Cell Signaling Technologies), anti-phospho c-Jun N-terminal kinase (JNK) Ab, total JNK Ab (both Cell Signaling Technologies), anti-NeuN Ab (Chemicon), anti-synaptophysin Ab (Dako), anti Actin Ab (Millipore), and anti-α-tubulin Ab (Sigma) as described earlier [24, 25].

Activity of p38 MAPK and JNK was estimated using commercially available in vitro immunocomplex protein kinase assays following the supplier’s instructions (Cell Signaling Technologies). The assays were standardized for equal volumes of lysate, beads for precipitation, indicator substrate (ATF-2 or c-Jun), elution, SDS-PAGE, and Western blotting procedure. Following immunoprecipitation, the kinase activities of p38 and JNK were detected using recombinant ATF-2 or c-Jun, respectively, as indicator substrates. Phosphorylated assay substrates (ATF-1 and c-Jun) were detected using an immunoblot-based non-radioactive system employing phosphorylation site-specific antibodies for visualization, and densitometry was used for quantification as recently described [24].

### Statistical analysis

Comparisons of more than two experimental groups were subjected to analysis of variance (ANOVA) followed by Fisher’s PLSD post hoc test. Analysis of two experimental conditions used student’s *t* test. Statistical analysis was performed with the StatView software package (version 5.0.1, SAS Institute, Cary, NC).

## Results

### CXCL12 compromises neuronal survival in a concentration-dependent fashion

**Fig. 1:**
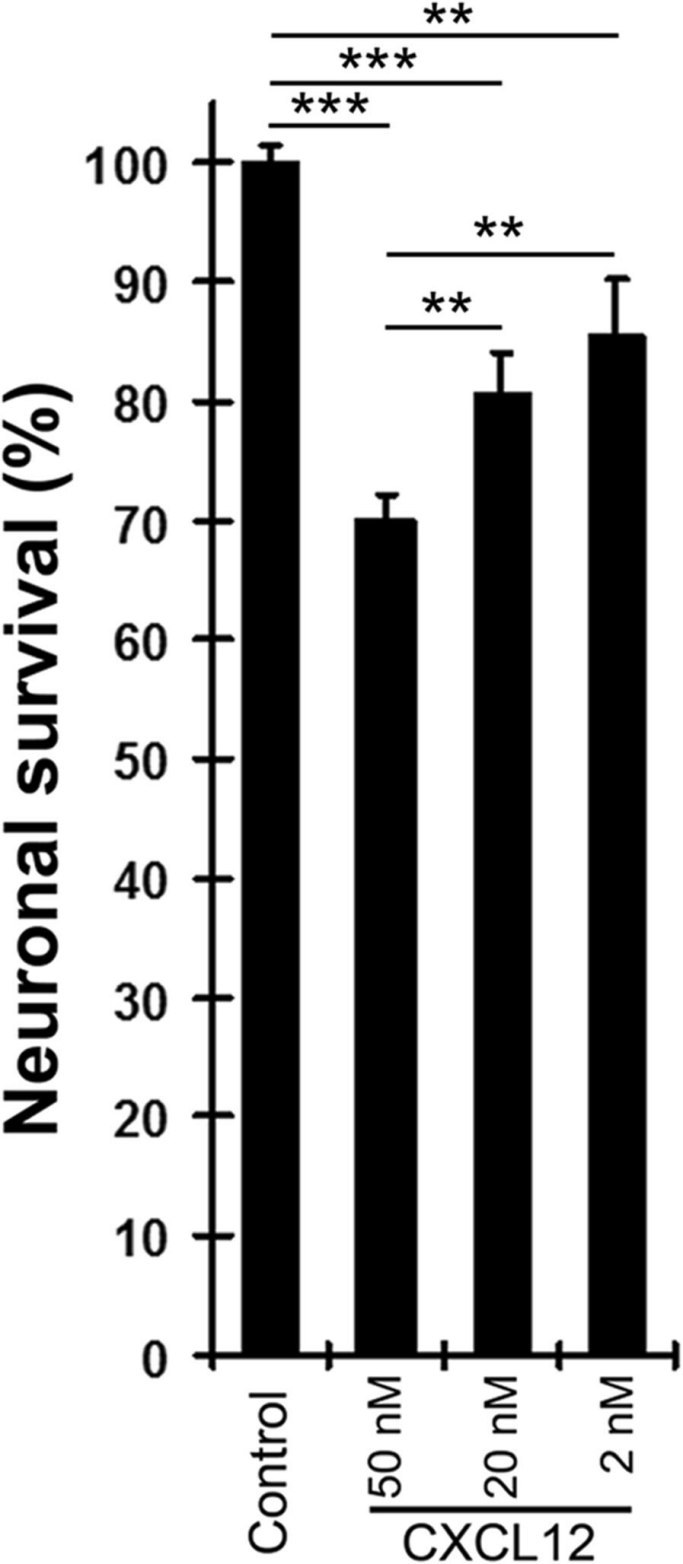
CXCL12 compromises neuronal survival in a concentration-dependent fashion. Mixed neuronal-glial cerebrocortical cell cultures were incubated for 24 h with CXCL12 at concentrations of 2, 20, and 50 nM. BSA (0.001 % final concentration) served as vehicle control in the absence of CXCL12. Assessment of neuronal survival was performed as described in the text using cell counting after fluorescence staining for neuronal MAP-2 and nuclear DNA. Values are mean ± SEM; *n* ≥ 3 with duplicate or triplicate samples per condition; **P ≤ 0.01; ***P ≤ 0.001 by ANOVA followed by Fisher’s PLSD post hoc test

First, the mixed neuronal-glial cerebrocortical cells were incubated for 24 h with CXCL12 at three different concentrations, 2, 20, and 50 nM. BSA (0.001 % final concentration) served as vehicle control in the absence of CXCL12. Following the incubation, the cell cultures were fixed, permeabilized, and fluorescence labeled for neuron-specific microtubule-associated protein-2 (MAP-2) and nuclear DNA as described in the “Methods” section. Comparing cell numbers and the percentages of neurons between the experimental conditions showed that CXCL12 reduced neuronal survival in a concentration-dependent fashion, Fig. 1, thus expanding our earlier observations using apoptosis and survival assays [12, 13].

### Blockade of Ca^2+^ channels prevents neurotoxicity of CXCL12

**Fig. 2:**
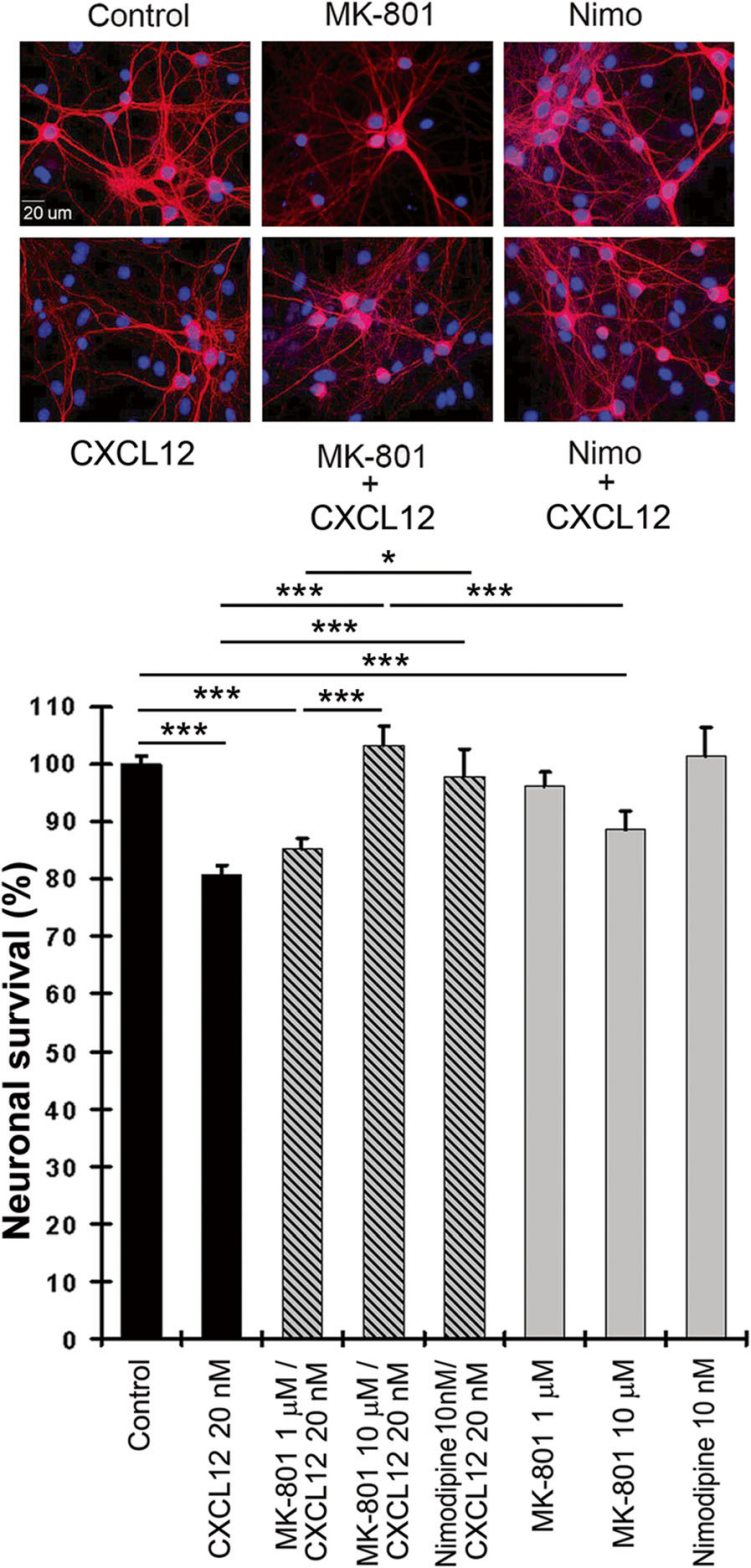
Ca^2+^ channel blockers MK-801 and nimodipine abrogate neurotoxicity of CXCL12 in cerebrocortical cell cultures. Incubation with CXCL12 and Ca^2+^ channel blockers and assessment of neuronal survival was performed as described in the text using staining for neurons (MAP-2) and nuclear DNA. Representative images are shown for six experimental conditions (MK-801 at 10 μM). *Scale bar*, 20 μm. Values in the graph are mean ± SEM; *n* ≥ 3 with duplicate or triplicate samples per condition; *P ≤ 0.05, ***P ≤ 0.001 by ANOVA followed by Fisher’s PLSD post hoc test

We previously showed that a neurotoxic concentration of CXCL12 triggers a transient increase of intracellular Ca^2+^ in cerebrocortical neurons [13]. Therefore, we next incubated cerebrocortical cells for 24 h with 20 nM of CXCL12 in the presence or absence of the NMDAR antagonist MK-801 (1 and 10 μM) or the l-type Ca^2+^ channel inhibitor nimodipine (10 nM). DMSO (0.1 %) served as vehicle control in the absence of MK-801 and nimodipine and BSA (0.001 %) as protein carrier control in the absence of CXCL12. The intermediate neurotoxic concentration of CXCL12 was chosen since it was initially unknown if the Ca^2+^ channel blockers would promote or prevent neurotoxicity of the CXCR4 ligand. The concentrations of MK-801 and nimodipine were derived from earlier reports of their protective effect against neurotoxicity of HIV-1 envelope protein gp120 [17]. Subsequent assessment of neurotoxicity showed that MK-801 at 10 μM and nimodipine at 10 nM completely abrogated neurotoxicity of CXCL12, Fig. 2. Interestingly and in contrast to its combination with CXCL12, MK-801 alone exerted a slight, yet significant, toxic effect at 10 μM concentration. However, MK-801 at 1 μM concentration was not toxic but also failed to protect neurons from CXCL12 toxicity. Nimodipine alone lacked any detectable influence on neuronal survival.

### CXCL12 activates p38 MAPK in cerebrocortical neurons

**Fig. 3:**
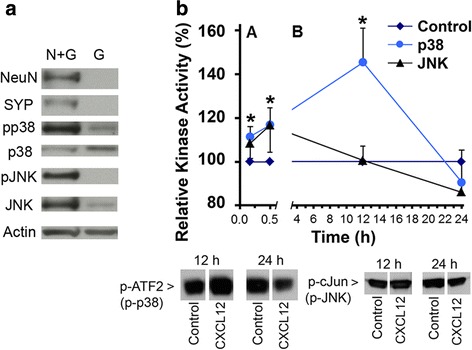
Neurotoxic CXCL12 transiently activates p38 MAPK and JNK in rat cerebrocortical cultures. **a** Complete neuro-glial cell cultures and neuron-depleted cerebrocortical cell cultures were analyzed by Western blotting for the presence of active p38 MAPK and JNK. To obtain glial cells, neurons were depleted by treating cerebrocortical cultures with 300 μM NMDA for 20 min 2 days prior to the preparation of cell lysates. Equal amounts of cellular protein (30 μg) were separated by SDS-PAGE and analyzed by immunoblotting for the indicated proteins. A representative Western blot from one of the three independent neuron depletion experiments is shown. **b** Cerebrocortical cultures were incubated with recombinant CXCL12 (20 nM) for the indicated time periods, prior to cell lysis on ice. Equal amounts of cellular protein (100 μg) were used for the performance of immunocomplex kinase assays as described in the “Methods” section. The Western blots show representative samples of phosphorylated indicator substrates observed with samples of the 12 and 24 h time points. Kinase activity in CXCL12-exposed samples and vehicle-treated controls was measured for each time point. Vehicle controls were defined as the 100 % baseline value. Note the difference in the dimension of time on the split *X*-axis. Each time point represents four to seven assessments in duplicate or triplicate in independent experiments. *P ≤ 0.05 compared to control by student’s *t* test. ‘pp38’/‘p-p38’ and ‘p-JNK’ indicate phosphorylated p38 MAPK and JNK, respectively

Using immunofluorescence microscopy, we previously found that in cerebrocortical cell cultures, active, phosphorylated p38 MAPK is primarily located to neurons and microglia, both of which, respectively, make up about 30 % and less than 1 % of the total cerebrocortical cell population [24]. Here, we compared cell lysates of complete neuro-glial (N+G) and neuron-depleted, glial cerebrocortical cultures (G) by Western blotting. Immunodetection of NeuN and synaptophysin was used to detect the presence and absence of neurons in N+G and G samples, respectively. We found that with this approach, phosphorylated p38 MAPK was strongly detectable in complete neuron-containing cerebrocortical cells, but not neuron-depleted glial cultures, which comprise about 98.6 % astrocytes and about 1.4 % microglia, Fig. 3a. This finding was consistent with the earlier observation that activation of p38 MAPK is predominantly detected in cerebrocortical neurons, which contribute the majority of kinase activity detected in immunocomplex kinase assays [24]. However, inactive, non-phosphorylated kinase is also found in glial cells. Surprisingly, the active, phosphorylated form of a second stress-related protein kinase, JNK, was predominantly detected in cerebrocortical cultures containing neurons and even the inactive, non-phosphorylated form of the kinase was only weakly detectable in glial cells.

Although neurons have high baseline activity of p38 MAPK compared to astrocytes and our studies had implicated the protein kinase in CXCL12 neurotoxicity, the activation kinetic of p38 MAPK in CXCL12-exposed cerebrocortical cells was previously unknown. Therefore, we first performed immunocomplex kinase assays using cell lysates harvested 10 and 30 min and 12 and 24 h after addition of CXCL12 or vehicle. For comparison, we also analyzed the activity of a second stress-related protein kinase, JNK. Figure 3b shows that, compared with the controls, p38 MAPK activity was consistently and significantly increased at 10 and 30 min and most pronounced 12 h exposure to CXCL12, while at 24 h, the level of kinase activity in CXCL12-exposed samples was on average below, yet not significantly distinct from the controls. The activity of JNK in the presence of CXCL12 did not significantly differ from control at any of the assessed time points, Fig. 3b.

**Fig. 4:**
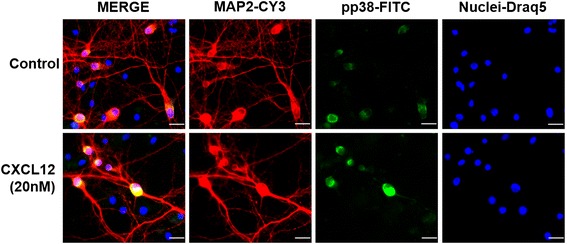
Phosphorylated p38 MAPK in cerebrocortical cell cultures localizes to neurons with and without CXCL12 exposure. Cerebrocortical cell cultures from rats were incubated with CXCL12 (20 nM). After 12 h of treatment, cells were fixed, permeabilized, and stained for MAP-2 (*red*), as a neuronal marker, activated-phospho p38 MAPK (*green*), and nuclear DNA (DRAQ5; shown in *blue pseudocolor* for better visualization). Samples were analyzed using immunofluorescence microscopy as described in the “Methods” section. The overlap of *red* and *green* signals appears *yellow* in the merged images. *Scale bars*, 20 μm

In additional experiments, we exposed mixed neuronal-glial cerebrocortical cell cultures for 12 h to 20 nM CXCL12 or vehicle control and subsequently fixed, permeabilized, and labeled the cells for phosphorylated p38 MAPK and neuronal MAP-2 as described in the “Methods” section. Immunofluorescence microscopy showed that phosphorylated p38 MAPK was primarily detected in neurons and with increased fluorescence intensity after exposure to CXCL12, Fig. 4.

### Inhibition of Ca^2+^ channels counteracts CXCL12-induced activation of p38 MAPK

**Fig. 5:**
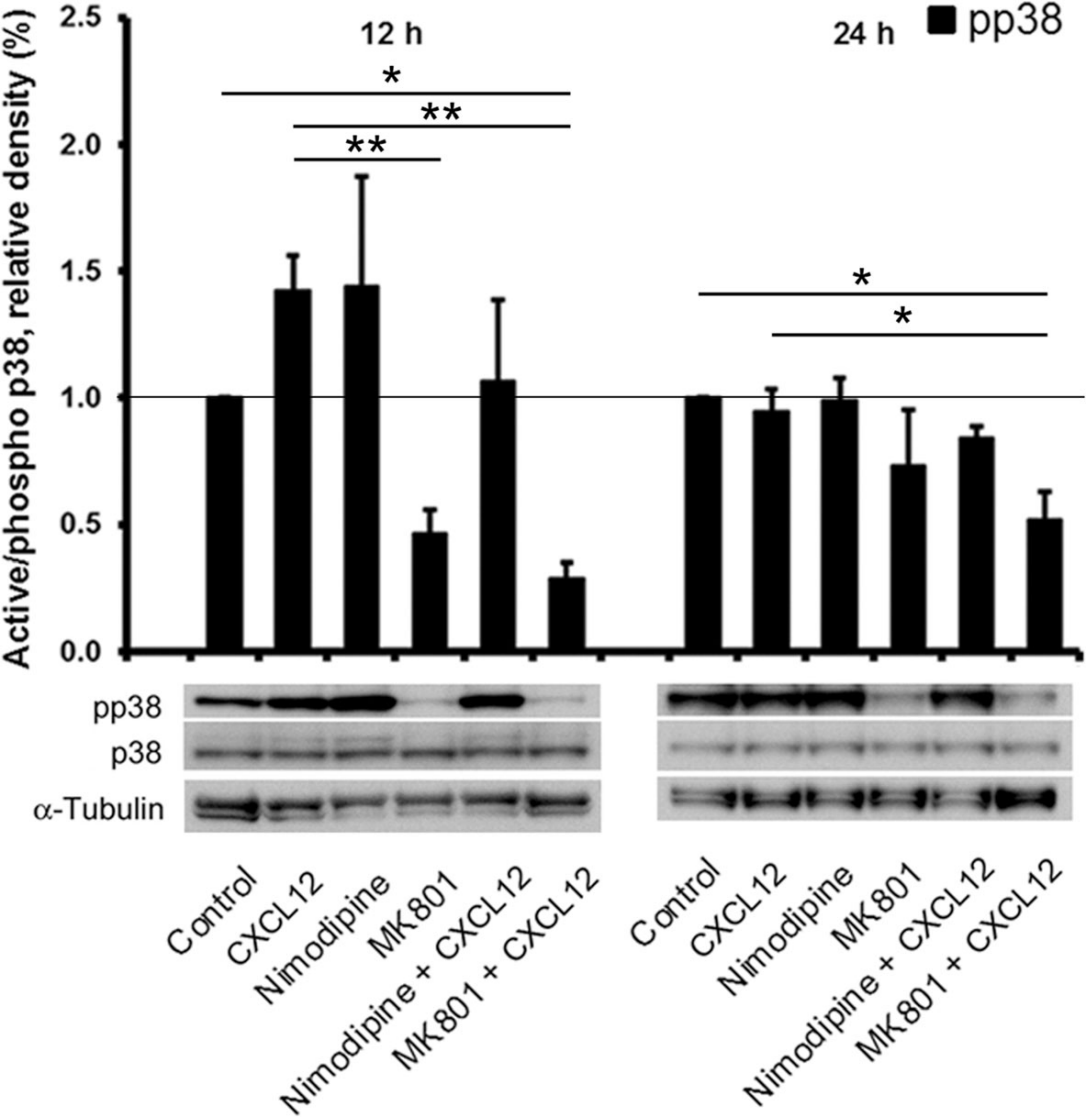
Ca^2+^ channel blockers limit activity of p38 MAPK in cerebrocortical cells upon exposure to CXCL12. Rat cerebrocortical cells were incubated for the indicated time periods with CXCL12 (20 nM) in the presence or absence of MK-801 (10 μM) or nimodipine (10 nM), prior to cell lysis on ice. 30 μg of protein per lane were analyzed by Western blotting using the specific antibodies against the indicated molecules. A representative immunoblot is shown for each time point. Quantification by densitometry; *n* = 3 to 4 per time point; *P ≤ 0.05, **P ≤ 0.01 by ANOVA followed by Fisher’s PLSD post hoc test

Since activation of p38 MAPK in neurons is influenced by intracellular Ca^2+^ levels [21] and we had previously shown that CXCL12 induces a transient increase of intracellular Ca^2+^ levels in neurons [13], we hypothesized that the Ca^2+^ channel blockers might limit neuronal death-inducing activation of the kinase by CXCL12. Therefore, we next employed Western blotting to estimate active p38 MAPK in cell lysates of cerebrocortical cells incubated for 12 and 24 h with a neurotoxic amount of CXCL12 (20 nM) in the presence or absence of MK-801 (10 μM) or nimodipine (10 nM). As before, DMSO (0.1 %) served as vehicle control in the absence of MK-801 and nimodipine, and BSA (0.001 %) as protein carrier control in the absence of CXCL12, Fig. 5. Detection of active/phosphorylated p38 MAPK by immunoblotting confirmed, besides the presence of active p38 MAPK under baseline conditions, the activity pattern upon CXCL12 exposure detected with the kinase assay at 12 and 24 h. Moreover, the immunoblotting experiments revealed that MK-801, but not nimodipine, significantly reduced the activation of p38 MAPK at 12 and 24 h. However, in the presence of nimodipine, the levels of active p38 MAPK did not differ significantly from the control upon CXCL12 exposure.

**Fig. 6:**
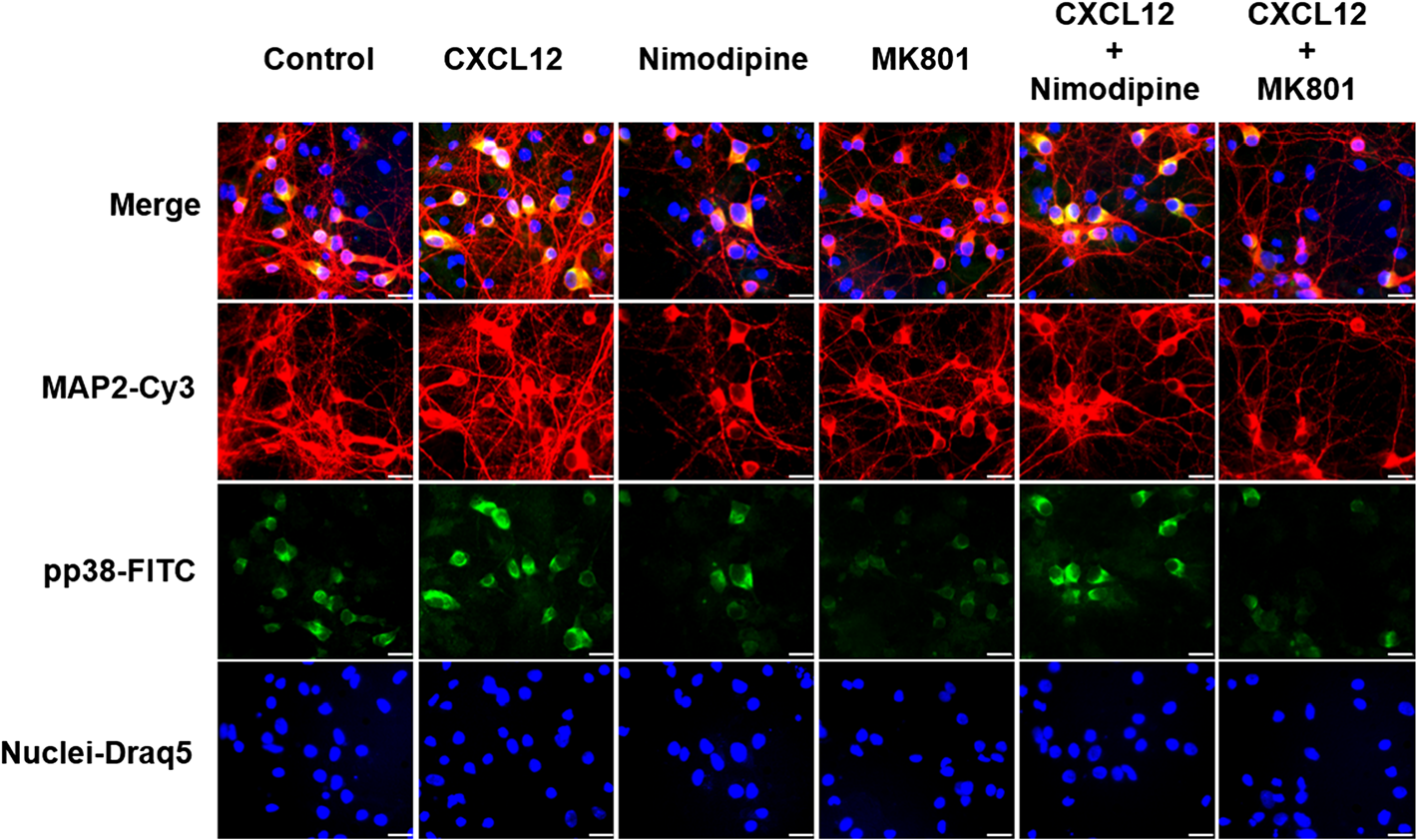
Phosphorylated p38 MAPK localization in neurons in the presence of CXCL12 and Ca^2+^ channel blockers. Cerebrocortical cell cultures were exposed to CXCL12 (20 nM), nimodipine (10 nM), or MK801 (10 μM) or combinations thereof for 12 h. Afterwards, cells were fixed, permeabilized, and stained for MAP-2 (*red*), activated/phospho p38 MAPK (*green*) and nuclear DNA (DRAQ5; shown in *blue pseudocolor* for better visualization). Images were analyzed using fluorescence microscopy as described in the “Methods” section. The overlap of *red* and *green* signals appears *yellow* in the merged images. *Scale bars*, 20 μm

In separate experiments, we treated mixed neuronal-glial cerebrocortical cell cultures for 12 h with 20 nM CXCL12 in the presence or absence of 10 μM MK-801 or 10 nM nimodipine. Controls received vehicle or one of the Ca^2+^ channel inhibitors without CXCL12. Following incubation, the cells were fixed, permeabilized, and labeled for phosphorylated p38 MAPK and neuronal MAP-2 as described in the “Methods” section. Analysis by immunofluorescence microscopy confirmed the location of phosphorylated p38 MAPK in neurons. Moreover, fluorescence intensity for phosphorylated p38 MAPK appeared increased by exposure to CXCL12 and diminished in the presence of MK-801, but not nimodipine, with and without CXC12, Fig. 6. Thus, the immunofluorescence microscopy of phosphorylated p38 MAPK supported the findings in the Western blotting experiments.

### Inhibition of p38 MAPK protects cerebrocortical neurons from CXCL12 toxicity

**Fig. 7:**
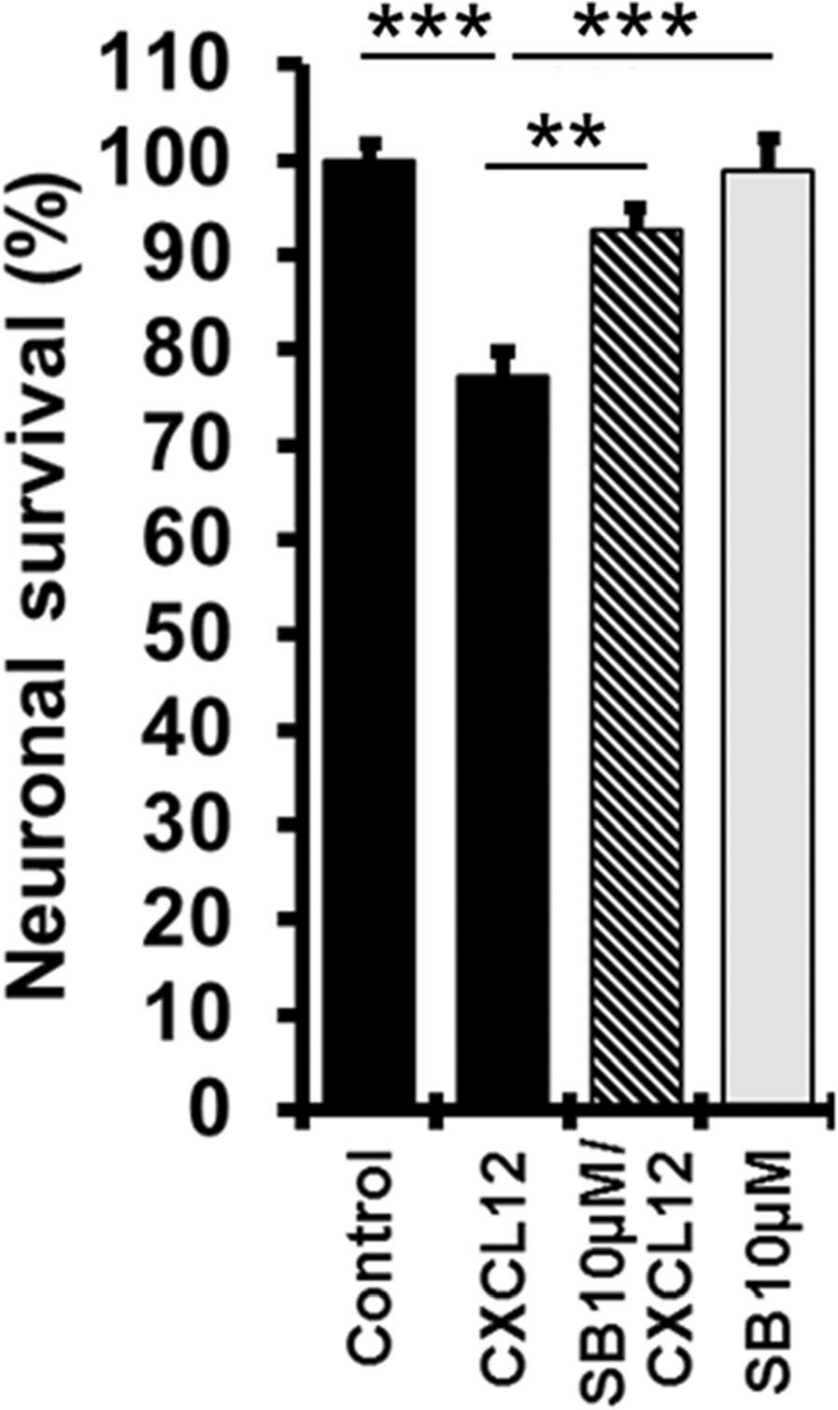
Pharmacological inhibition of p38 MAPK protects cerebrocortical neurons from toxicity of CXCL12. Cerebrocortical cell cultures were incubated with CXCL12 (20 nM) in the presence and absence of p38 MAPK inhibitor SB203580 (SB, 10 μM) for 24 h. Analysis of neuronal survival was performed as described in the “Methods” section using staining for neurons (MAP-2) and nuclear DNA (H33342). Values are mean ± SEM; *n* ≥ 3 with duplicate or triplicate samples per condition; **P ≤ 0.01, ***P ≤ 0.001 by ANOVA followed by Fisher’s PLSD post hoc test

In analogy to the experiment with the Ca^2+^ channel blockers, we incubated cerebrocortical cells for 24 h with 20 nM of CXCL12 in the presence or absence of the p38 MAPK inhibitor SB203580 (SB, 10 μM). DMSO (0.1 %) served as vehicle control in the absence of SB203580 and BSA (0.001 %) as protein carrier control in the absence of CXCL12. Neuronal survival was assessed by fluorescence microscopy and cell counting as described in the “Methods” section after the cell cultures were fixed, permeabilized, and fluorescently labeled for neuron-specific microtubule-associated protein-2 (MAP-2) and nuclear DNA. Comparison of neuronal cell numbers between the experimental conditions confirmed that inhibition of p38 MAPK prevented CXCL12 from inducing significant neurotoxicity, Fig. 7.

## Discussion

In the CNS, the functions of CXCR4 and CXCL12 include beyond cell migration also the regulation of development, axonal outgrowth and patterning, synaptic function, and plasticity as well as decisions about cell death and survival (reviewed in [4–6, 8, 9, 31–35]). We confirmed recently that CXCL12 is constitutively expressed in the murine CNS [30] and demonstrated in vitro that about 93 % of cerebrocortical cells, virtually all neurons and microglia, and 88 % of astrocytes expressed CXCR4 protein [13]. Moreover, we showed in vitro that CXCL12 triggered a transient increase of intracellular Ca^2+^ levels in cerebrocortical neurons [13].

Earlier, we found that full-length CXCL12 is toxic to terminally differentiated cerebrocortical neurons via a pathway that depended on CXCR4 and implicated p38 MAPK [12, 13, 36]. Others linked neurotoxic CXCR4 activation to astrocytic release of glutamate that is stimulated via a pathway comprising TNFα, prostaglandins, and required microglial activation [19]. In addition, potassium channels have recently been shown to play a role in CXCR4-mediated induction of neuronal death [37]. Here, we showed that blockade of NMDAR-gated ion channels and l-type Ca^2+^ channels both prevent CXCR4-mediated toxicity of CXCL12 in cerebrocortical neurons and thus established a link between the CXCL12 induced neuronal death and cellular Ca^2+^ regulation. Excessive Ca^2+^ influx via NMDAR occurs in neuronal excitotoxicity [17, 38]. Therefore, our results indicated a link between the injurious effect of CXCL12 and excitotoxicity, a finding which was in line with the report by Bezzi and colleagues that CXCL12 triggered astrocytic glutamate release [19].

Since we had previously shown that CXCL12 induces a transient increase of intracellular Ca^2+^ levels in neurons [13] and others demonstrated that activation of p38 MAPK in neurons is influenced by intracellular Ca^2+^ levels [21], we wondered if CXCL12 significantly increased in cerebrocortical neurons the baseline activity of the p38 MAPK. Our experiments confirmed that CXCL12 transiently increased activity of neuronal p38 MAPK, but not JNK, significantly above baseline. In contrast, p38 MAPK activity was strongly reduced by blocking neuronal NMDAR with MK-801 and kept overall similar to control levels by blocking l-type Ca^2+^ channels with nimodipine. Thus, while inhibition of l-type Ca^2+^ channels did not suppress p38 MAPK activity significantly below the baseline, it still prevented a significant increase which seems to be required for induction of neuronal death [24].

While Ca^2+^-permeable NMDAR are primarily expressed in neurons, l-type Ca^2+^ channels are present in neuronal and glial cells, namely astrocytes and microglia [39–42]. However, voltage-sensitive l-type Ca^2+^ channels and NMDAR-gated ion channels provide the main entry sites for Ca^2+^ into neurons [42]. Similar to what has been observed for l-type Ca^2+^ channels, we found that about 93 % of cerebrocortical cells, neurons and glia, express CXCR4 protein [13]. Considering the reported involvement of astrocytes and microglia in CXCL12-induced glutamate release and toxicity, it seemed probable that nimodipine, in contrast to MK-801, exerted its neuroprotective effect through interaction with all cerebrocortical cell types. Future studies will aim at distinguishing what the contribution of glial versus neuronal l-type Ca^2+^ channels is in the response to CXCL12.

In a recent study, investigating neurotoxicity triggered by HIV-1 envelope protein gp120, we found that active p38 MAPK primarily resided in neurons [24], which is in line with the pronounced effect of NMDA receptor blockade by MK-801 on the kinase’s activity in the present study. Furthermore, Ca^2+^ influx via NMDAR-gated ion channels has been shown to activate neuronal p38 MAPK and excitotoxic death [21] and thus fits with our findings that CXCL12-induced neuronal cell death is associated with a transient increase in neurons of intracellular Ca^2+^ [13] and of p38 MAPK activity, as shown here with kinase assays, Western blotting, and immunofluorescence.

**Fig. 8:**
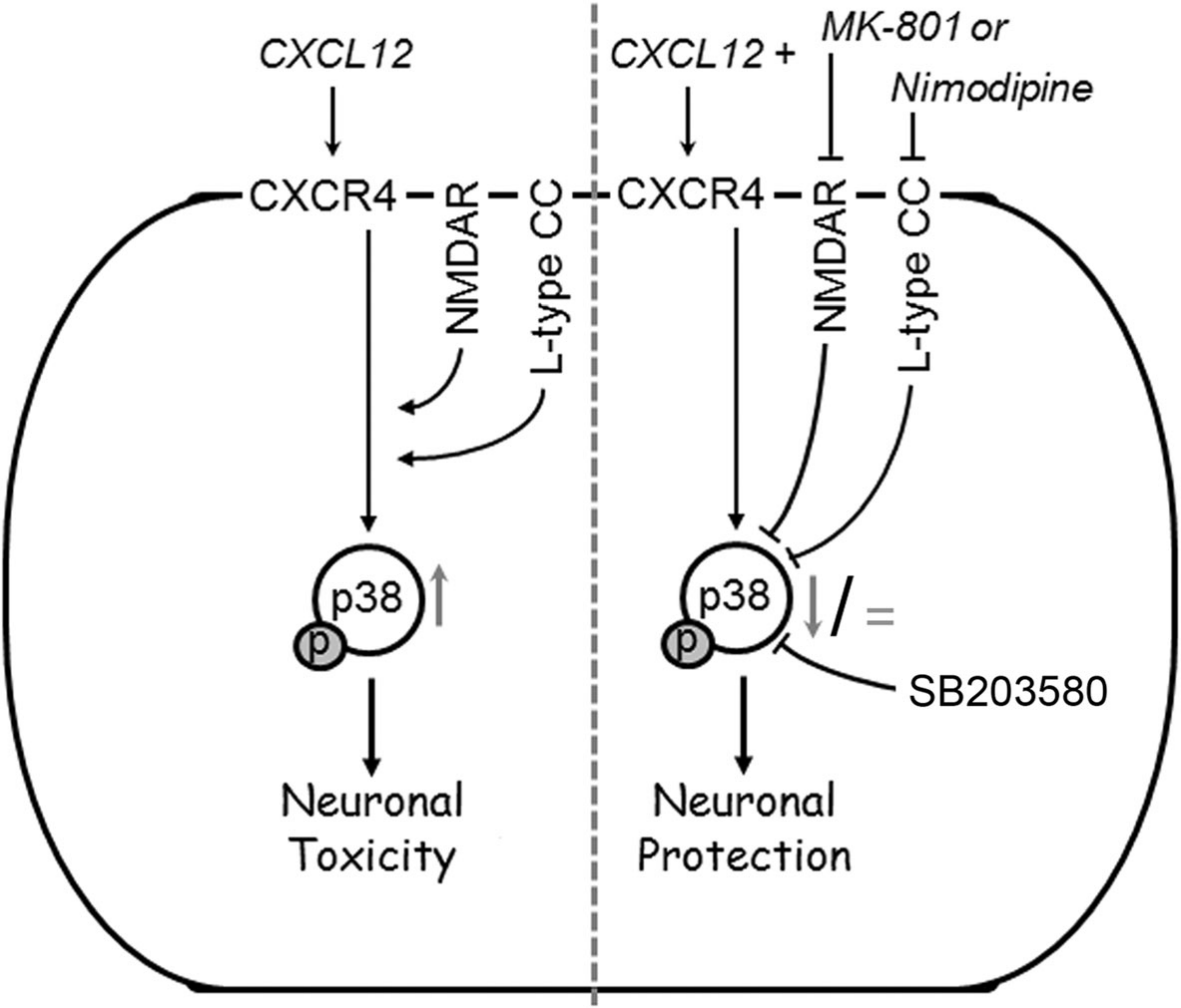
NMDA receptors and l-type Ca^2+^ channels can regulate CXCL12 induced neuronal death upstream of p38 MAPK activation. Activity of p38 MAPK (phosphorylation indicated by *p*) increases above baseline levels (*upward arrow*) as a critical mediator of CXCR4-mediated neurotoxicity of CXCL12. The present study implicates besides NMDAR-gated ion channels for the first-time l-type Ca^2+^ channels (l-type CC) in CXCL12 neurotoxicity as the blockade of both independently can prevent CXCL12 neurotoxicity while concomitantly preventing an increase (nimodipine; *equal sign*) or reducing (MK-801; downward arrow) the activity of p38 MAPK compared to baseline

Moreover, our confirmation that direct pharmacological blockade of p38 MAPK can prevent CXCL12 neurotoxicity supports the notion that limiting or diminishing the activity of the stress kinase in neurons by inhibition of l-type Ca^2+^ channels and NMDAR, respectively, is a critical component of the neuroprotective mechanism. Hence, our findings indicate that both types of Ca^2+^ channels can control CXCL12-induced neuronal death upstream of p38 MAPK. Figure 8 summarizes our findings in a schematic representation.

The best recognized pathological function of CXCR4 is its role as a co-receptor during infection with HIV-1 [10]. Furthermore, we and others have shown that CXCR4 can mediate the induction of neuronal injury and apoptosis by HIV-1 envelope protein gp120 [12–16, 30, 43]. The neurotoxic process downstream of chemokine receptor engagement by HIV-1 or its envelope protein gp120 also involves excessive excitatory activation of the NMDAR (excitotoxicity) which in turn can be blocked by the same Ca^2+^ channel inhibitors that we used in the present study [17]. On the other hand, elevated levels of CXCL12 mRNA have been reported for brain specimen from HIV encephalitis (HIVE) patients as compared to uninfected controls [16, 44], and we and others have shown that protein expression of CXCL12 also appears to be elevated in the brains of HIV patients [45, 46]. Macrophages infected with HIV-1 have been suggested to induce astrocytosis via CXCL12 and matrix metalloproteinases [47], and HIV-1-infected as well as immune activated macrophages have been reported to regulate astrocyte CXCL12 production through IL-1β [48]. All these observations suggest the possibility that under pathological conditions, such as HIV infection, an increase of CXCL12 concentrations beyond physiological levels could contribute to neuronal injury.

However, recently published data suggested two seemingly opposing scenarios regarding the function of the CXCR4-CXCL12 axis during HIV infection. First, the CXCR4-CXCL12 interaction can promote neurocognitive impairment and CNS injury in association with HIV infection in the same way described in the present study [12, 13, 36, 46]. On the other hand, a different set of studies proposed that the same receptor-ligand system could ameliorate HIV-induced brain injury by supporting neuronal survival [14, 49]. The two scenarios are not necessarily exclusive, because one possible explanation is that the overall effect of the CXCR4-CXCL12 interaction depended on its exact context, such as the composition, type, and age of the neuronal cell culture or, in vivo, the specific brain region where the receptor-ligand system was engaged.

Our present study revealed that the combination of 20 nM CXCL12 with 10 μM MK-801 lacked any neurotoxicity, whereas CXCL12 and MK-801 each killed a significant number of neurons when applied separately at the same concentrations. Toxicity of MK-801 towards cerebrocortical neurons and microglia has previously been observed but was found to be preventable in both cell types in the presence of glutamate [50–53]. MK-801 indiscriminately blocks synaptic and extra-synaptic NMDAR but physiological Ca^2+^ influx through synaptic NMDAR triggers survival signals in neurons [54]. Thus, the absence of neurotoxicity in cerebrocortical cells under the condition of combined exposure to CXCL12 and MK-801 suggested that not only MK-801 blocked CXCL12’s detrimental effect but that CXCL12 might paradoxically in turn have protected neurons in a situation where a neuronal survival-threatening blockade of NMDARs by MK-801 occurred. However, this hypothetical explanation requires further investigation.

## Conclusions

Stimulation of CXCR4 by the chemokine CXCL12 can induce toxicity in cerebrocortical neurons in a concentration-dependent fashion. CXCL12 induces an increase of neuronal p38 MAPK activity that is counteracted by blockade of NMDAR-gated and l-type Ca^2+^ channels. Pharmacological inhibition of NMDAR-gated and l-type Ca^2+^ channels as well as p38 MAPK each abrogates neurotoxicity of CXCL12. Hence, our study indicates that NMDAR-gated and l-type Ca^2+^ channels are in addition to p38 MAPK critical components of the neurotoxic mechanism induced by CXCL12. Moreover, our findings suggest that both types of Ca^2+^ channels can control CXCL12-induced neuronal death upstream of p38 MAPK.

## Abbreviations

Ab: Antibody
BCA: Bicinchoninic acid
CXCL: CXC motif chemokine ligand
CXCR: CXC motif chemokine receptor
DMSO: Dimethyl sulfoxide
HIV: Human immunodeficiency virus
JNK: c-Jun N-terminal kinase
MAPK: Mitogen-activated protein kinase
NMDAR: NMDA-type glutamate receptor-gated ion channel
PBS: Phosphate-buffered saline
PVDF: Polyvinyl difluoride
SDF: Stromal cell-derived factor
TNF: Tumor necrosis factor
